# Psychometric properties of the Teachers’ Sense of Efficacy Scale in a sample of Chilean public school teachers

**DOI:** 10.3389/fpsyg.2023.1272548

**Published:** 2023-09-22

**Authors:** José Luis Gálvez-Nieto, Sonia Salvo-Garrido, Sergio Domínguez-Lara, Karina Polanco-Levicán, Manuel Mieres-Chacaltana

**Affiliations:** ^1^Departamento de Trabajo Social, Universidad de La Frontera, Temuco, Chile; ^2^Departamento de Matemática y Estadística, Universidad de La Frontera, Temuco, Chile; ^3^South American Center for Education and Research in Public Health, Universidad Privada Norbert Wiener, Lima, Peru; ^4^Programa de Doctorado en Ciencias Sociales, Universidad de La Frontera, Temuco, Chile; ^5^Departamento de Diversidad y Educación Intercultural, Universidad Católica de Temuco, Temuco, Chile

**Keywords:** efficacy, teachers, validity, reliability, measurement invariance

## Abstract

The Teachers’ Sense of Efficacy Scale (TSES) has demonstrated suitable levels of reliability and validity for its use on the teacher population in several countries, and it is the most used scale to assess teachers’ beliefs in their efficacy. However, few psychometric studies exist on its applicability to elementary teachers in public schools. This study analyzed the psychometric properties of the TSES in teachers who work in elementary education. The sample comprised 1,406 Chilean teachers, mainly women (77.2%), from various Chilean public and subsidized schools. The results obtained from three confirmatory factor analyses demonstrated that the model that best fit the data was bifactor exploratory structural equation modeling (B-ESEM) for 24 items, one general factor, and three residual factors. The results of the factorial invariance analysis indicate that the TSES remains stable up to the strict level of invariance for the variable sex. These results imply that the TSES can be used on Chilean teachers. The results are discussed based on the theoretical and empirical evidence available.

## 1. Introduction

Teacher self-efficacy is considered fundamental to students’ teaching-learning processes, since it favors the quality of education, especially elementary (Lazarides et al., 2020; Opoku et al., 2022; Yin, 2022). As a result, there has recently been an increase in interest in its assessment, particularly at a time when schools are dealing with complex circumstances that make it difficult for them to achieve their goals (Guangbao and Timothy, 2021). In the case of Chile, there is a structural problem posed by high socioeconomic-based segregation, which has affected students’ development of different skills and academic performance and has also affected their families (Murillo and Garrido, 2017; Murillo et al., 2018, 2023; Salvo-Garrido et al., 2020). As a result, the work of teachers has been hampered and, therefore, challenged; even more so if the management of favorable learning environments depends largely on the talent and self-efficacy of the teachers (Bandura, 1995). Consequently, a successful response to the challenge of teaching in conditions like those described would require a greater sense of teacher self-efficacy (Murillo et al., 2018; Yang et al., 2021).

Bandura (1981, 1984) states that self-efficacy refers to the judgments a person makes about themself and their opportunities to flexibly organize their cognitive, social, and behavioral skills, taking actions that enable them to face situations with positive results. It is important to mention that perceived self-efficacy affects a person’s behavior, thinking, and emotions, leading them to choose different courses of action, thereby influencing the activities and environments in which they develop (Bandura, 1981). This occurs in changing, unpredictable, or stressful contexts; therefore, the performance of a person’s skills requires improvisation (Bandura, 1982, 1984). In particular, the perception of self-efficacy influences the effort invested in a particular task and the persistence that the person shows considering the appearance of obstacles (Bandura, 1984).

In this sense, teacher self-efficacy is understood as teachers’ beliefs about the performance of their skills that would allow them to carry out the proposed tasks successfully, achieving the goals of the class (Tschannen-Moran and Woolfolk Hoy, 2001; Dellinger et al., 2008; Hoang and Wyatt, 2021). Teacher self-efficacy focuses on how a person behaves and reflects on responding to the demands of the environment (Downes et al., 2021), and it is related to the confidence and competences displayed by the teacher when participating in an activity, considering that their actions can lead their students to positive outcomes (Lemon and Garvis, 2016). In the same vein, teachers’ pedagogical practices that show greater self-efficacy focus on the success of their students; in addition, they are accessible and benefit the boy or girl’s confidence. By contrast, those teachers with low self-efficacy focus on student behavior management (Woodcock et al., 2022).

With respect to teacher self-efficacy, it is worth noting that it is related to aspects linked to the teaching-learning process, such as teacher autonomy (Nguyen et al., 2023), with skills in managing elementary students in the classroom (Lazarides et al., 2020), and that it is negatively associated with anxiety due to teaching mathematics (Bosica, 2022). Also, it is associated with work-related aspects: job satisfaction, teacher commitment, and emotional intelligence (Granziera and Perera, 2019; Sökmen and Sarikaya, 2022). Therefore, teachers with higher levels of self-efficacy show greater job commitment and less exhaustion (Burić and Macuka, 2018; Fathi et al., 2021; Shu, 2022), are involved in specialized training activities, learning and using new teaching strategies (Kent and Giles, 2017; Shu, 2022). In this sense, these teachers show greater effort in reaching their goals despite the difficulties they face (Burić and Macuka, 2018). On the other hand, a high perception of self-efficacy is related to higher socioemotional competences, specifically in women between 40 and 50 years old and teachers with more experience (Romero-García et al., 2022). In addition, they more frequently experience joy, pride, and love, and less anger, fatigue, and hopelessness toward their students (Burić and Macuka, 2018).

However, teacher self-efficacy is important for students to accomplish the expected learning outcomes in a given course and achieve a suitable academic performance, including subjects like English (Murillo et al., 2018; Mahmoodi et al., 2022). In addition, teacher self-efficacy favors the teacher-student relationship (Granziera and Perera, 2019; Shu, 2022), as this is directly associated with proximity and inversely with conflict (Zee et al., 2017; Hajovsky et al., 2020; Wettstein et al., 2021), as well as with the reduction in problems with externalizing behavior (Finch et al., 2023). Considering the diversity of students in classrooms, it is observed that teachers experience a lower perception of self-efficacy teaching students with disabilities (Guo et al., 2021), demonstrating that teacher self-efficacy is fundamental since it favors inclusive education in schools (Yada et al., 2018; Opoku et al., 2022), improving the teaching-learning process in students with attention-deficit hyperactivity disorder and those with learning difficulties (Chunta and DuPaul, 2022).

Against this backdrop, Tschannen-Moran and Woolfolk Hoy (2001) proposed the Teachers’ Sense of Efficacy Scale (TSES), which comprises three dimensions. The first is Efficacy in instructional strategies, which refers to the teacher’s perception of their ability to develop various strategies according to the needs of the class, respond appropriately to students’ questions, and provide adequate challenges for the most advantaged students while also taking into account the appropriate assessment of the contents covered, all to help their students perform their best. The second is called Efficacy in classroom management. This factor accounts for the teacher’s ability to get a student to conform to classroom rules, supporting emotional and behavioral management so all students learn. The last one, Efficacy in student engagement, refers to the teacher’s ability to have students develop their activities adequately, showing confidence and valuing their own abilities while supporting the family in the educational process (Tschannen-Moran and Woolfolk Hoy, 2001). It is important to note that, in the case of Chile, there are regulatory provisions clearly linked to the abilities mentioned in the TSES and which are required of teachers. These are stated in the Framework for Good Teaching (Ministerio de Educación de Chile, 2021).

It is important to mention that the TSES presents evidence of validity and reliability in studies in Latin America. Dominguez-Lara et al. (2019) performed the adaptation and validation of the instrument in a sample of Peruvian teachers in urban public schools and reported a bifactor structure with 24 items, which indicates that one global factor best explains the variance of the items compared to the specific factors. On the other hand, Salas-Rodríguez et al. (2021) conducted a study with Mexican elementary and secondary school teachers, and although they maintain that the TSES is best represented by a factor structure of three correlated factors that coincides with the original scale, the interfactor correlations were high (>0.80; Brown, 2015), which would make one wonder if a global factor is present (Flores-Kanter et al., 2018). Therefore, more studies in Latin America are required to evaluate the psychometric properties of this widely used scale in different parts of the world to assess teaching effectiveness (Salas-Rodríguez et al., 2021).

In Chile, Covarrubias-Apablaza and Mendoza-Lira (2016) adapted the TSES to a sample of teachers in public, subsidized, and private urban schools in regions in central Chile (Viña del Mar and Valparaiso). Their results yielded a factor structure of four dimensions with 17 items, i.e., one more dimension than the original version of the scale that contains three factors with 24 items. The first three are: Efficacy in instructional practices, Efficacy in classroom management, and Efficacy in student engagement; this is to say, in line with the authors’ proposal of the scale (Tschannen-Moran and Woolfolk Hoy, 2001). The fourth factor is “Efficacy in addressing student uniqueness” (Covarrubias-Apablaza and Mendoza-Lira, 2016). This addresses teachers’ ability to teach to the diversity of students in the classroom. It is important to mention that the study in question did not report the criteria for determining the number of factors, method of estimation and rotation (exploratory analysis), or the factor loadings and interfactor correlations; as a result, there is no clarity or relevance to the structure evaluated using the confirmatory factor analysis, which affects the estimation of reliability. There is also no consistent theoretical foundation to account for the name of the fourth dimension, nor does the new proposal overcome the problem of under-representation produced by shifting items from other dimensions to this additional dimension.

Regarding the type of school where the teachers work, Covarrubias-Apablaza and Mendoza-Lira (2016) refer to teachers who work in private schools having a greater perception of self-efficacy, whereas it decreases in teachers who work in public schools. Contrastingly, Pérez Norambuena et al. (2023) describe public school teachers as showing greater self-efficacy. In addition, they report that the perception of self-efficacy in physical education teachers is generally high, decreasing the efficacy factor in classroom management. On the other hand, teachers in public and subsidized schools and those who work in preschools report a greater ability to address student uniqueness (Covarrubias-Apablaza and Mendoza-Lira, 2016). In addition, there is evidence of a relationship between greater teacher efficacy and greater professional experience, and a teacher’s efficacy in generating appropriate strategies for their students to learn is linked to postgraduate teacher training compared to those who only have undergraduate training (Covarrubias-Apablaza and Mendoza-Lira, 2016; Pérez Norambuena et al., 2023).

It is worth noting that both Covarrubias-Apablaza and Mendoza-Lira (2015) and Pérez Norambuena et al. (2023) have reported that there are no significant differences between Chilean teachers of different genders in their perception of self-efficacy, which is endorsed by several international studies (Sirmaci and Taş, 2016; Tárraga-Mínguez et al., 2022). In their studies, Covarrubias-Apablaza and Mendoza-Lira (2015) mention that men and women teachers feel able to engage, teach, manage, and care for their students.

As a result of the above, considering the empirical and theoretical relevance of the Teacher Self-Efficacy construct, it is necessary to provide more evidence about its factor structure in the population of teachers working in elementary education, given that this has not yet been clarified in the Chilean context. Therefore, this study aimed to analyze the psychometric properties of the TSES in Chilean teachers who work in elementary education in public schools.

## 2. Method

### 2.1. Design

The study is based on an instrumental design, where the psychometric properties of the TSES for adults were studied (Ato et al., 2013).

### 2.2. Participants

The study population comprises all the teachers who work in the elementary education in public and subsidized schools in Chile, *N* = 85,298. A stratified random sample was selected considering the following strata: region, habitat (urban, rural), type of education, and sex. Stratified, multistage probability sampling was used, with a reliability of 95%, a sampling error of 2.5%, and a variance *p* = *q* = 0.5 (Scheaffer et al., 1987). The sample comprised *n* = 1,406 teachers, women (77%) and men (22.5%), other (0.3%), and prefer not to say (0.2%), with a mean age of 41.43 years (SD = 10.84).

### 2.3. Instruments

A sociodemographic questionnaire collected information on the teacher’s age, gender, ethnic group, type of school, and sector in the workplace, among others.

The Teachers’ Sense of Efficacy Scale (TSES). The TSES is a self-report scale that measures teachers’ perceptions of self-efficacy (Tschannen-Moran and Woolfolk Hoy, 2001) based on 24 items answered on a five-point ordinal scale (1 = nothing, 5 = a great deal). This scale has a structure of three correlated factors called: Efficacy in instructional strategies (8 items, e.g., “To what extent can you craft good questions for your students?”), Efficacy in classroom management (8 items, e.g., “How much can you do to get children to follow classroom rules?”), and Efficacy in student engagement (8 items, e.g., “How much can you do to help your students value learning?”). The original study that proposes this instrument provides evidence of the psychometric quality of validity and reliability (Tschannen-Moran and Woolfolk Hoy, 2001).

### 2.4. Procedures

All public school principals, mayors, and directors of Local Education Services were contacted because, in Chile, public schools are under the administration of municipalities, and Local Education Services are under the Chilean Ministry of Education. The study was presented to all these actors, and their authorization was sought so the schools under their purview could participate.

The data collection was carried out via a computerized platform (Question Pro), which contained the TSES, a questionnaire with sociodemographic questions, and the informed consent, which explained the aim of the study, the voluntary nature of the study, risks and benefits, among others, to protect the ethical principles of the project. Visits were scheduled for application in the schools to ensure the sample size. The study was approved by the Scientific Ethics Committee of the Universidad de La Frontera, Chile.

### 2.5. Data analysis

First, the descriptive statistics of each of the items were analyzed. Then, the measurement models were evaluated with exploratory structural equation modeling (*ESEM*; Asparouhov and Muthén, 2009) using the *MPLUS* v.8.1 software (Muthén and Muthén, 2017). A polychoric correlations matrix and the estimation method of weighted least squares means and variance adjusted (*WLSMV*) was used considering the ordinal nature of the variables and the absence of normality (Hancock and Mueller, 2001). The teachers’ responses to the TSES were modeled considering the evidence of the initial psychometric study, a model of three correlated factors (Tschannen-Moran and Woolfolk Hoy, 2001). Furthermore, following the final results of a Chilean study (Covarrubias-Apablaza and Mendoza-Lira, 2016), a model with 17 items distributed in four correlated factors was implemented. Finally, according to the results of a psychometric study in Peru (Dominguez-Lara et al., 2019), a bifactor exploratory structural equation model (*B-ESEM*) was estimated with the 24 items on the TSES. This model comprised a general factor of teacher self-efficacy and three specific factors referring to the dimensions of Efficacy in instructional strategies, Efficacy in classroom management, and Efficacy in student engagement.

In the case of the estimations of the *ESEM* models, target rotation was used, which makes it possible to use this technique in confirmatory mode as it produces the rotated solution closest to a prespecified loading configuration (Asparouhov and Muthén, 2009). This provides a more robust model *a priori* and facilitates the interpretation of the results (Marsh et al., 2014). For the evaluation of *CFA* models, the following goodness-of-fit indices were used: *WLSMV-χ^2^*, comparative fit index (*CFI*), Tucker-Lewis index (*TLI*), and root mean square error of approximation (*RMSEA*). For the *CFI* and *TLI*, values greater than or equal to 0.96 were considered reasonable (Hair et al., 2019). For the *RMSEA*, values less than or equal to 0.08 were considered reasonable (Browne and Cudeck, 1993). Additionally, factor loadings greater than 0.50 in the theoretical factor were considered acceptable (Dominguez-Lara, 2018), and the factor simplicity index (*FSI*; Fleming and Merino, 2005) greater than 0.70 indicated that the item is predominantly influenced by a single factor (Dominguez-Lara and Merino-Soto, 2018; Lara et al., 2021).

To evaluate the unidimensionality of the scale under bifactor modeling, explained common variance (*ECV*), percentage of uncontaminated correlations (*PUC*), and percentage of reliable variance (*PRV*) were used (Brunner et al., 2012; Reise, 2012; Ríos and Wells, 2014; Bonifay et al., 2015). For interpretation purposes, values of *ECV* > 0.70 and *PUC* > 0.70 indicate slight relative bias, and the common variation can be considered essentially unidimensional (Rodríguez et al., 2015). If the *PUC* exceeds 0.80, the *ECV* values are less influential in predicting bias (Reise et al., 2013). Concerning the *PRV*, values over 75% indicate strong evidence for using the score from a subscale. Additionally, the omega hierarchical (*ωh*) and Omega hierarchical subscale (*ωhs*) were considered (Zinbarg et al., 2005; Reise et al., 2013; Rodríguez et al., 2015). The first more accurately estimates the strength of a general factor in structural equation models (Gignac, 2014, 2015), and values over 0.75 indicate the predominance of only one general factor (Reise et al., 2013), in addition to offering a clear contrast with the weight of the specific factors (Brunner et al., 2012). The *ωhs* evaluates the variance explained by each specific dimension controlling for the presence of the general factor, and magnitudes over 0.30 indicate that it is possible to interpret the specific factor (Smits et al., 2015).

The reliability score was estimated with Cronbach’s alpha coefficient (*α*) (Cronbach, 1951), and the construct reliability with McDonald’s omega (*ω*) (McDonald, 1999) and *H* index (Hancock and Mueller, 2001). *H* is a measure of construct replicability which “represent[s] the correlation between a factor and an optimally-weighted item composite. Then, high *H* values (>0.80) suggest a well-defined latent variable” (Hancock and Mueller, 2001, p. 230).

In addition, a factorial invariance analysis was performed, which includes the following models (Vandenberg and Lance, 2000): M0 configural (equal number of factors), M1 weak (equal factor loadings), M2 strong (equality of thresholds), and M3 strict (equality of residuals). Invariance was evaluated following the recommendations by Chen (2007) based on the following criteria: *ΔCFI* ≤ 0.001 and *ΔRMSEA* ≤ 0.015 as evidence of invariance.

## 3. Results

### 3.1. Descriptive analysis

Next, the descriptive results of the items on the scale appear (Table 1). As observed, item 6, “How much can you do to get students to believe they can do well in school work?” had the highest mean (*M* = 4.33, *SD* = 0.769). In contrast, item 22, “How much can you assist families in helping their children do well in school?” had the lowest mean (*M* = 3.76, *SD* = 0.955). Univariate normality was also estimated; the results provided by the Kolmogorov–Smirnov test make it possible to reject the null hypothesis of normality (*p* < 0.001). The multivariate kurtosis test was also estimated, yielding results consistent with the univariate tests, rejecting the hypothesis of multivariate normality (multivariate kurtosis coefficient = 150.635, *p* < 0.001).

**Table 1:** Descriptive statistics.

Items	M	SD	g1	g2	K-S test
Item 1	4.02	0.825	−0.516	−0.216	0.242*
Item 2	4.0	0.819	−0.447	−0.341	0.242*
Item 3	3.89	0.873	−0.352	−0.587	0.228*
Item 4	4.08	0.829	−0.492	−0.507	0.223*
Item 5	4.1	0.81	−0.627	0.017	0.237*
Item 6	4.34	0.771	−0.923	0.190	0.311*
Item 7	4.18	0.763	−0.603	−0.113	0.238*
Item 8	4.14	0.802	−0.562	−0.340	0.234*
Item 9	4.23	0.795	−0.696	−0.254	0.272*
Item 10	4.13	0.805	−0.606	−0.155	0.229*
Item 11	4.11	0.793	−0.559	−0.198	0.233*
Item 12	4.19	0.811	−0.671	−0.231	0.254*
Item 13	4.17	0.819	−0.690	−0.137	0.251*
Item 14	3.97	0.862	−0.524	−0.201	0.237*
Item 15	3.95	0.857	−0.452	−0.382	0.239*
Item 16	4.05	0.833	−0.564	−0.157	0.236*
Item 17	3.99	0.882	−0.534	−0.376	0.227*
Item 18	4.02	0.885	−0.613	−0.177	0.226*
Item 19	3.88	0.93	−0.552	−0.204	0.236*
Item 20	4.25	0.806	−0.892	0.373	0.274*
Item 21	3.91	0.897	−0.525	−0.207	0.239*
Item 22	3.77	0.959	−0.464	−0.344	0.231*
Item 23	4.04	0.858	−0.613	−0.058	0.231*
Item 24	4.08	0.831	−0.620	−0.088	0.233*

M, Mean; SD, Standard Deviation; g1, Skewness; g2, Kurtosis; *p < 0.001.

### 3.2. Confirmatory factor analysis

To assess the factorial structure of the TSES, three confirmatory factorial models were evaluated considering previous psychometric studies. The first model tested was the one with 24 items and three correlated factors. This model provided unsatisfactory goodness-of-fit indicators *WLSMV-χ^2^* (*df* = 249) = 1899.139, *p* < 0.001; *CFI* = 0.913; *TLI* = 0.903; *RMSEA* = 0.069 (90% *CI* = 0.066–0.072). The second model evaluated consisted of 17 items and four correlated factors. This model also provided unsatisfactory goodness-of-fit indicators *WLSMV-χ^2^* (*df* = 113) = 901.104, *p* < 0.001; *CFI* = 0.935; *TLI* = 0.921; *RMSEA* = 0.070 (90% *CI* = 0.66–0.75). Finally, the bifactor exploratory structural equation modeling of 24 items was the model that obtained satisfactory indicators of goodness of fit *WLSMV-χ^2^* (*df* = 276) = 98479.596, *p* < 0.001; *CFI* = 0.988; *TLI* = 0.981; *RMSEA* = 0.069 (90% *CI* = 0.065–0.072). Considering these results, it is possible to conclude that the model that best fitted the data is the bifactor exploratory structural equation modeling of 24 items.

Table 2 presents the standardized factor loadings and the indices calculated to assess the reliability and unidimensionality of the TSES. The loadings in the general factor of TSES were statistically significant and presented a range that varied between 0.906 and 0.677. All the indicators used to assess the unidimensionality of the scale exceeded their cut-off scores, for example, *ECV* for the general factor was 0.880, *ωh* of the general factor was 0.959, *PRV* of the general factor was 94.4%, which indicates strong evidence in favor to use the total score. In contrast, all *PRV* values for the subscales were well below the 75% cutoff. This evidence contradicts the use of the scores of some of them.

**Table 2:** Standardized factor loadings resulting from the bifactor-ESEM model and indicators of unidimensionality and reliability of the TSES.

Items	Theoretical factor	FG	F1	F2	F3
Item 1	F1	0.696 (0.02)*	0.440 (0.02)*	0.014 (0.02) ns	0.115 (0.02)*
Item 2	F1	0.726 (0.01)*	0.400 (0.02)*	−0.029 (0.02) ns	−0.049 (0.02)*
Item 4	F1	0.843 (0.01)*	0.264 (0.02)*	−0.041 (0.01)*	0.095 (0.02)*
Item 6	F1	0.889 (0.01)*	0.184 (0.02)*	−0.078 (0.02)*	−0.049 (0.02)*
Item 9	F1	0.906 (0.01)*	0.057 (0.02)*	−0.066 (0.01)*	−0.048 (0.01)*
Item 12	F1	0.857 (0.01)*	**−0.007 (0.02)** ns	0.035 (0.02)*	−0.101 (0.02)*
Item 14	F1	0.837 (0.01)*	−0.056 (0.02)*	0.075 (0.02)*	0.094 (0.01)*
Item 22	F1	0.677 (0.02)*	**0.020 (0.03)** ns	0.25 (0.02)*	0.272 (0.02)*
Item 7	F2	0.837 (0.01)*	0.084 (0.02)*	**−0.024 (0.02)** ns	−0.095 (0.02)*
Item 10	F2	0.895 (0.01)*	−0.107 (0.02)*	**0.026 (0.02)** ns	−0.116 (0.01)*
Item 11	F2	0.889 (0.01)*	−0.118 (0.02)*	0.058 (0.02)*	−0.209 (0.02)*
Item 17	F2	0.809 (0.01)*	−0.030 (0.01)*	0.367 (0.02)*	0.079 (0.01)*
Item 18	F2	0.753 (0.01)*	−0.075 (0.02)*	0.451 (0.02)*	0.011 (0.01) ns
Item 20	F2	0.834 (0.01)*	−0.030 (0.02) ns	0.232 (0.02)*	0.017 (0.01) ns
Item 23	F2	0.802 (0.01)*	0.042 (0.01)*	0.462 (0.02)*	0.038 (0.01)*
Item 24	F2	0.809 (0.01)*	0.026 (0.02) ns	0.379 (0.02)*	0.048 (0.01)*
Item 3	F3	0.744 (0.01)*	0.219 (0.02)*	−0.156 (0.02)*	0.38 (0.02)*
Item 5	F3	0.843 (0.01)*	0.202 (0.02)*	−0.120 (0.01)*	0.109 (0.02)*
Item 8	F3	0.877 (0.01)*	0.019 (0.02) ns	−0.090 (0.01)*	**0.007 (0.02)** ns
Item 13	F3	0.865 (0.01)*	−0.071 (0.02)*	−0.126 (0.01)*	0.182 (0.02)*
Item 15	F3	0.796 (0.01)*	−0.005 (0.02) ns	0.008 (0.01) ns	0.448 (0.02)*
Item 16	F3	0.870 (0.01)*	−0.071 (0.01)*	0.078 (0.01)*	0.23 (0.02)*
Item 19	F3	0.736 (0.01)*	−0.076 (0.02)*	0.152 (0.01)*	0.468 (0.02)*
Item 21	F3	0.753 (0.01)*	0.019 (0.02) ns	0.175 (0.02)*	0.364 (0.02)*
	ECV	0.88	0.026	0.042	0.044
	*α*	0.972			
	*ω*	0.985			
	ωh	0.959			
	ωhs		0.037	0.077	0.098
	*H*	0.982	0.354	0.477	0.489
	PUC	0.697			
	PRV	97.4	3.9	8.0	1.2

ECV, Explained Common Variance; *α*, Cronbach’s alpha coefficient; *ω*, Coefficient omega; ωh, Omega Hierarchical; ωhs, Omega Hierarchical Subscale; *H*, Index H; PUC, Percentage of Uncontaminated Correlations; PRV, Percentage of Reliable Variance. Values in bold indicate factor loadings in the primary dimension. Values in parentheses correspond to the standard error. *p < 0.001; ns, non-statistically significant.

Regarding the evidence of reliability (Table 2), the omega coefficient presented a value equal to 0.985 and the alpha coefficient 0.972 for the general factor. The *H* index of the general factor was 0.982, which means that it is a well-defined one-dimensional latent variable.

### 3.3. Measurement invariance

Once the factorial structure of the TSES was obtained, a factorial invariance analysis for the variable sex was performed (1 = Woman, 0 = Man). The first model contrasted was the one of configural invariance M0; the results were satisfactory (Table 3) and make it possible to conclude that the factor structure of the TSES is the same for men and women. Then, a model of weak invariance (M1) that imposes restrictions on the factor loadings was evaluated. The results indicated no significant variations between the fit indices of the weak and configuration models; therefore, the factor loadings of the items on the scale are equivalent according to sex. In addition, the degree of strong invariance (M2) was evaluated, including restrictions on the thresholds of the items, and the results indicate that the indices of fit did not vary substantially between the weak and strong models, which indicates that the thresholds of the items of men and women are statistically similar. Finally, a model of strict invariance (M3) was evaluated, which imposes restrictions on the residuals, and the results indicate that there are no significant differences between the indices of fit of the strong and strict models according to sex, concluding that the residuals of the items appear in similar magnitudes between men and women.

**Table 3:** Factorial invariance according to sex.

Model	*RMSEA* (CI90%)	*CFI*	*TLI*	*WRMR*	*ΔCFI*	*ΔTLI*	*ΔRMSEA*
M0	0.048 (0.045–0.052)	0.993	0.991	1.274			
M1	0.042 (0.039–0.045)	0.994	0.993	1.388	0.001	0.002	−0.006
M2	0.040 (0.037–0.043)	0.994	0.994	1.406	0	0.001	−0.002
M3	0.039 (0.036–0.043)	0.994	0.994	1.594	0	0	−0.001

RMSEA, root mean square error of approximation; CFI, comparative fit index; TLI, Tucker–Lewis index; WRMR, Weighted Root Mean Square Residual.

## 4. Discussion

The purpose of this article was to analyze the psychometric properties of the TSES in Chilean teachers working in elementary education in public schools. The results obtained in this study show the total achievement of this objective by providing solid evidence of the validity and reliability of the scale used in the context of Chilean teachers.

In this study, three factor models were assessed. The first model considered the theoretical structure proposed in the original psychometric study, which included 24 items distributed across three correlated factors (Tschannen-Moran and Woolfolk Hoy, 2001). In addition, following the results of a previous Chilean psychometric study (Covarrubias-Apablaza and Mendoza-Lira, 2016), a CFA was implemented with 17 items and a structure of four correlated factors. Finally, following the results of a Peruvian study (Dominguez-Lara et al., 2019), a bifactor B-ESEM model was implemented, which best fit the data and presented satisfactory goodness-of-fit indices and adequate reliability.

The results obtained from the bifactor B-ESEM model supported the factor structure comprised of one general factor and three latent factors and are consistent with the study conducted in Peru (Dominguez-Lara et al., 2019). The general factor explained the overall trait of teachers’ sense of efficacy, referring to their perception of their ability to effectively carry out their role-related tasks and responsibilities (Tschannen-Moran and Woolfolk Hoy, 2001). The three specific factors represented the components of the characteristic related to Efficacy in instructional strategies, Efficacy in classroom management, and Efficacy in student engagement.

The findings obtained through models of factorial invariance support the equivalence of the Teacher Self-efficacy Scale (TSES) up to the level of strict invariance based on sex. The results in the sample indicate that the TSES measures men and women teachers equivalently. These results align with the Peruvian study (Dominguez-Lara et al., 2019) that provided evidence of measurement invariance between men and women. In addition, two previous Chilean studies that found no significant differences in teacher self-efficacy factors according to sex can be taken into account (Covarrubias-Apablaza and Mendoza-Lira, 2015; Pérez Norambuena et al., 2023). Similar findings were reported in other countries (Sirmaci and Taş, 2016; Tárraga-Mínguez et al., 2022).

With respect to the limitations of this study, it is important to note that private school teachers did not participate. This omission is relevant considering that private schools represent at least 10% of the total enrolment in Chile (Ministerio de Educación de Chile, 2021). It is recommended that psychometric studies continue being conducted to provide evidence of convergent validity and to analyze the invariability of the construct in various teacher populations. This will facilitate the comparison of results between different sociocultural contexts and strengthen the robustness of the conclusions obtained in this study.

## Data availability statement

The raw data supporting the conclusions of this article will be made available by the authors, without undue reservation.

## Ethics statement

The studies involving humans were approved by Ethics Committee of Universidad de La Frontera (File N°053_21; Study Protocol Page N°019/21). The studies were conducted in accordance with the local legislation and institutional requirements. The participants provided their written informed consent to participate in this study. Written informed consent was obtained from the individual(s) for the publication of any potentially identifiable images or data included in this article.

## Author contributions

JG-N: Conceptualization, Data curation, Formal analysis, Methodology, Resources, Software, Supervision, Visualization, Writing – original draft, Writing – review & editing. SS-G: Conceptualization, Data curation, Formal analysis, Funding acquisition, Investigation, Methodology, Project administration, Resources, Software, Supervision, Validation, Visualization, Writing – original draft, Writing – review & editing. SD-L: Conceptualization, Formal analysis, Methodology, Resources, Software, Visualization, Writing – original draft. KP-L: Conceptualization, Data curation, Resources, Supervision, Visualization, Writing – original draft, Writing – review & editing. MM-C: Conceptualization, Data curation, Resources, Supervision, Visualization, Writing – review & editing.

## Funding

The author(s) declare financial support was received for the research, authorship, and/or publication of this article. This research was funded by Agencia Nacional de Investigación y Desarrollo: FONDECYT Projects 1210551.

## Conflict of interest

The authors declare that the research was conducted in the absence of any commercial or financial relationships that could be construed as a potential conflict of interest.

## Publisher’s note

All claims expressed in this article are solely those of the authors and do not necessarily represent those of their affiliated organizations, or those of the publisher, the editors and the reviewers. Any product that may be evaluated in this article, or claim that may be made by its manufacturer, is not guaranteed or endorsed by the publisher.
