# Supplementary material for: Psychometric properties of the Teachers’ Sense of Efficacy Scale in a sample of Chilean public school teachers
Source: Front Psychol. 2023 Sep 22;14:1272548. doi: 10.3389/fpsyg.2023.1272548 (PMC10556521; doi:10.3389/fpsyg.2023.1272548)
Supplement: Supplementary file 1 [file Table_1.DOCX]

Supplementary Material

Teachers' Sense of Efficacy Scale

| **Items** |
| --- |
| 1.- How much can you do to get through to the most difficult students? [1.- ¿Cuánto puede hacer usted para comunicarse con las y los alumnos más difíciles?] |
| 2.- How much can you do to help your students think critically? [2.- ¿Cuánto puede hacer usted para ayudar a las y los alumnos a pensar de manera crítica?] |
| 3.- How much can you do to control disruptive behavior in the classroom? [3.- ¿Cuánto puede hacer usted para controlar la conducta disruptiva en la sala de clases?] |
| 4.- How much can you do to motivate students who show low interest in school work? [4.- ¿Cuánto puede hacer usted para motivar a las y los alumnos que muestran poco interés en el trabajo escolar?] |
| 5.- To what extent can you make your expectations clear about student behavior? [5.- ¿Cuánto puede hacer usted para aclarar a las y los alumnos sus expectativas sobre el comportamiento?] |
| 6.- How much can you do to get students to believe they can do well in school work? [6.- ¿Cuánto puede hacer usted para que las y los alumnos crean que pueden realizar bien el trabajo escolar?] |
| 7.- How well can you respond to difficult questions from your students? [7.- ¿Cuánto puede hacer usted para responder a las preguntas difíciles que hacen las y los alumnos?] |
| 8.- How well can you establish routines to keep activities running smoothly? [8.- ¿Cuánto puede hacer usted para establecer rutinas a fin de mantener las actividades de las y los alumnos desarrollándose fluidamente?] |
| 9.- How much can you do to help your students value learning? [9.- ¿Cuánto puede hacer usted para ayudar a las y los alumnos a valorar el aprendizaje?] |
| 10.- How much can you gauge student comprehension of what you have taught? [10.- ¿Cuánto puede hacer usted por medir si las y los alumnos comprendieron lo que les ha enseñado?] |
| 11.- To what extent can you craft good questions for your students? [11.- ¿Cuánto puede hacer usted para elaborar buenas preguntas para las y los alumnos?] |
| 12.- How much can you do to foster student creativity? [12.- ¿Cuánto puede hacer usted para fomentar la creatividad de las y los alumnos?] |
| 13.- How much can you do to get children to follow classroom rules? [13.- ¿Cuánto puede hacer usted para que las y los alumnos sigan las normas de la sala de clases?] |
| 14.- How much can you do to improve the understanding of a student who is failing? [14.- ¿Cuánto puede hacer usted para mejorar la comprensión de las y los alumnos que están reprobando?] |
| 15.- How much can you do to calm a student who is disruptive or noisy? [15.- ¿Cuánto puede hacer usted para calmar a las y los alumnos que presentan comportamientos disruptivos o bulliciosos?] |
| 16.- How well can you establish a classroom management system with each group of students? [16.- ¿Cuánto puede hacer usted para establecer un sistema de manejo de la clase con cada grupo de alumnos?] |
| 17.- How much can you do to adjust your lessons to the proper level for individual students? [17.- ¿Cuánto puede hacer usted para ajustar las clases al nivel adecuado de cada una/o de las y los alumnos?] |
| 18.- How much can you use a variety of assessment strategies? [18.- ¿Cuánto puede hacer usted por utilizar diversas estrategias de evaluación?] |
| 19.- How well can you keep a few problem students from ruining an entire lesson? [19.- ¿Cuánto puede hacer usted para evitar que unos pocos alumnos(as) disruptivos perjudiquen toda la clase?] |
| 20.- To what extent can you provide an alternative explanation or example when students are confused? [20.- ¿Cuánto puede hacer usted para dar explicaciones o ejemplos adicionales cuando las y los alumnos están confundidos?] |
| 21.- How well can you respond to defiant students? [21.- ¿Cuánto puede hacer usted para responder a las y los alumnos confrontacionales?] |
| 22.- How much can you assist families in helping their children do well in school? [22.- ¿Cuánto puede hacer usted por asistir a las familias para que ayuden a las y los alumnos a desempeñarse bien en la escuela?] |
| 23.- How well can you implement alternative strategies in your classroom? [23.- ¿Cuánto puede hacer usted para implementar estrategias alternativas en la clase?] |
| 24.- How well can you provide appropriate challenges for very capable students? [24.- ¿Cuánto puede hacer usted por ofrecer desafíos apropiados para las y los alumnos con buen desempeño?] |

1= Not at all [Nada], 2 = A little [Poco], 3 = Quite a bit [Bastante], 4 = A lot [Mucho], 5 = Very much [Muchísimo]
